# Causality of gut microbiome and hypertension: A bidirectional mendelian randomization study

**DOI:** 10.3389/fcvm.2023.1167346

**Published:** 2023-05-04

**Authors:** Yihui Li, Ru Fu, Ruixuan Li, Jianwei Zeng, Tao Liu, Xiaogang Li, Weihong Jiang

**Affiliations:** Department of Cardiology, The Third Xiangya Hospital, Central South University, Changsha, China

**Keywords:** gut microbiome, hypertension, genome-Wide association study, causality, mendelian randomization

## Abstract

**Background & Aims:**

The pathogenesis of hypertension involves a diverse range of genetic, environmental, hemodynamic, and more causative factors. Recent evidence points to an association between the gut microbiome and hypertension. Given that the microbiota is in part determined by host genetics, we used the two-sample Mendelian randomization (MR) analysis to address the bidirectional causal link between gut microbiota and hypertension.

**Methods:**

We selected genetic variants (*P* < 1  ×  10^−5^) for gut microbiota (*n* = 18,340) from the MiBioGen study. Genetic association estimates for hypertension were extracted from genome-wide association study (GWAS) summary statistics on 54,358 cases and 408,652 controls. Seven complementary MR methods were implemented, including the inverse-variance weighted (IVW) method, followed by sensitivity analyses to verify the robustness of the results. Reverse-direction MR analyses were further conducted to probe if there was a reverse causative relationship. Bidirectional MR analysis then examines a modulation of gut microbiota composition by hypertension.

**Results:**

At the genus level, our MR estimates from gut microbiome to hypertension showed that there were 5 protective factors *Allisonella*, *Parabacteroide*, *Phascolarctobacterium*, *Senegalimassilia,* and *unknowngenus* (id.1000000073), while 6 genera *Clostridiuminnocuum*, *Eubacteriumcoprostanoligenes*, *Eubacteriumfissicatena*, *Anaerostipes*, *LachnospiraceaeFCS020*, and *unknowngenus* (id.2041) are risk factors. The *Alcaligenaceae* and *ClostridialesvadinBB60* were detrimental and beneficial at the family level, respectively. In contrast, the MR results of hypertension-gut flora showed hypertensive states can lead to an increased abundance of E*ubacteriumxylanophilum*, *Eisenbergiella*, and *Lachnospiraceae* and a lower abundance of *Alistipes*, *Bilophila*, *Butyricimonas*, and *Phascolarctobacterium*.

**Conclusion:**

Altered gut microbiota is a causal factor in the development of hypertension, and hypertension causes imbalances in the intestinal flora. Substantial research is still needed to find the key gut flora and explore the specific mechanisms of their effects so that new biomarkers can be found for blood pressure control.

## 1. Introduction

Hypertension is a worldwide problem with major health implications, and its incidence continues to rise with unsatisfactory control rates (1). High blood pressure (BP) is a primary risk factor for cardiovascular, cerebrovascular, and kidney disease (2–4). BP control is considerable, with even a 2-mmHg drop reducing mortality from cardiovascular disease by 7% (5). The etiology of hypertension concerns genetics and the complex interaction of environmental and pathophysiological factors affecting multiple systems, and the regulatory mechanism of BP has yet to be entirely elucidated (6–8). Researchers incidentally noted a relatively higher pressure level in mice with gut microbiota colonization compared to germ-free mice (9). Alternatively, fecal matter from hypertensive patients was transplanted into germ-free mice and an increase in BP was observed (10). Variations in intestinal flora can perhaps directly influence hypertension.

Over past decades, gut microbes have attracted intense attention from scientists for promising roles in altering human organismal health. The gut microbiota synthesizes and facilitates the uptake of key nutrients and ions, sculpts the mucosa's immune system, and produces multiple metabolites and chemical mediators for action (11–13). Host genetic variation intervenes in differences amongst individuals for gut microbiota composition, likewise environmental exposure (14, 15). The ecological dysbiosis of the intestinal microbiota is implicated in both intestinal and extraintestinal diseases, including hypertension (16, 17). Several cross-sectional analyses proved that changes in human BP correlate with intestinal flora. The BP showed significant separation based on the abundance of intestinal microorganisms, which exhibits a negative correlation (18). Alleviation of hypertension also appears to involve specific bacteria taxa such as those that produce short-chain fatty acids (SCFAs) (19, 20). Such microbial flora as, *Faecalibacterium*, *Ruminococcaceae Eubacterium rectale*, and *Roseburia* have a high butyrate production capacity, which contributes to the decrease in systolic BP (21). Crucially, taking probiotics improves hypertension to some extent (22). That means it is not just about the abundance of flora, a lack of healthy flora or dysbiosis may promote higher BP (23). However, studies of clinical hypertension causation are particularly challenging in that the major pathophysiological mechanisms may be obscured by confounding effects of compensatory pathways, diet, or medication, just as *Clostridiales* shows opposing effects in the different hypertensive population studies (24, 25). Even more importantly, dysbiosis may also be a concomitant symptom of hypertension. Larger animal studies or clinical cohorts are still required to identify the gut flora associated with hypertension and how they are causally connected.

We used Mendelian randomization (MR) analysis to establish a causal relationship between exposure and outcome achieved through an instrumental variable using genetic variations. Such an approach exploits the random classification of genetic variation during gamete formation, consequently minimizing the possible effects of confounding factors and reverse causality (26). Here, we performed bidirectional MR analyses of causal inference between hypertension and gut microbiota. Clues for potential hazards of hypertension can be found in the hypertension-regulated flora. What's more, this causal inquiry provides suspected biomarkers for further research into hypertension pathogenesis and therapeutic targets for better BP control.

## 2. Material & methods

### 2.1. Two-Sample bidirectional MR design

**Figure 1:**
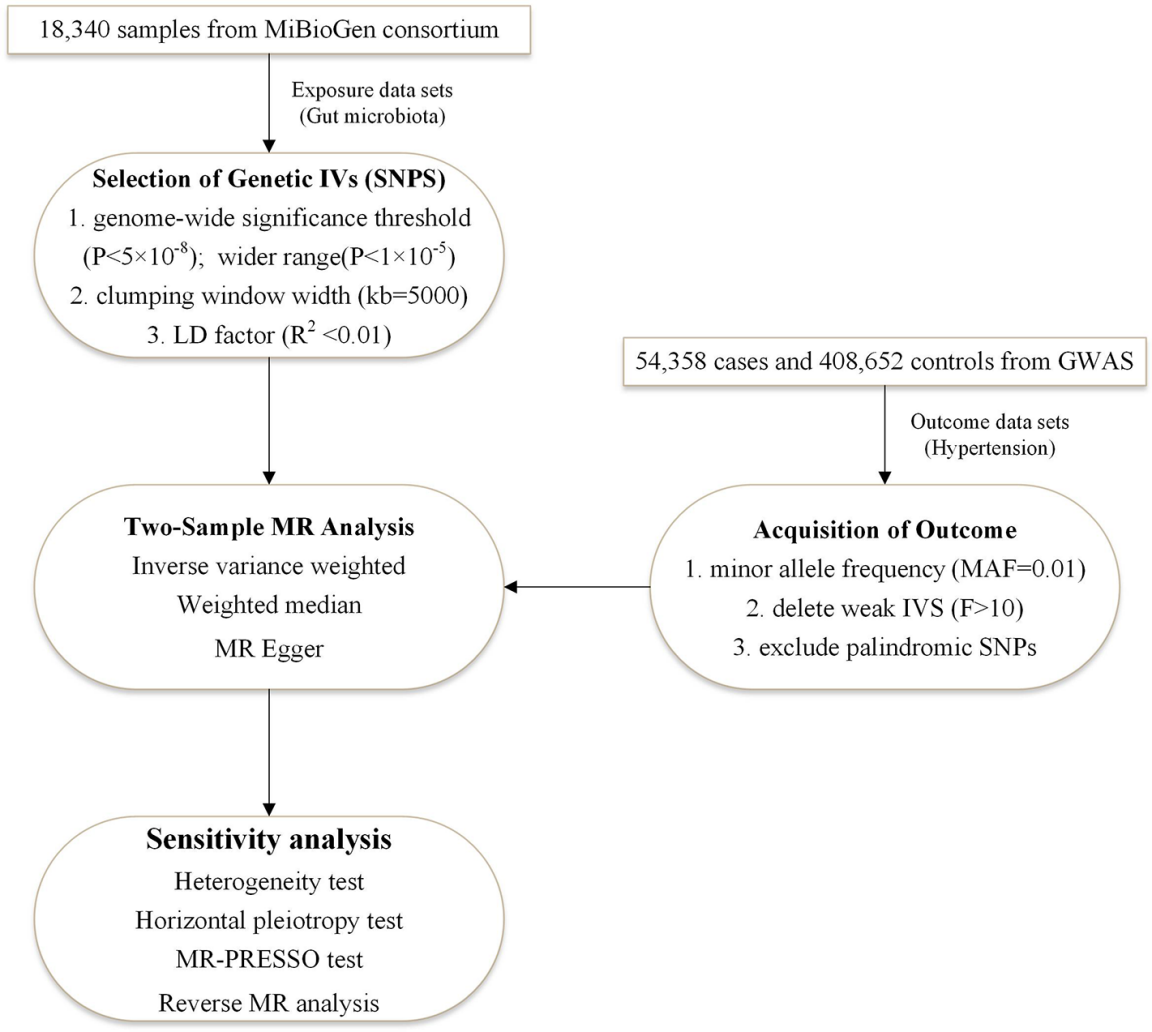
The two-sample MR-analysis process. Furthermore, we performed a bidirectional MR analysis in the same way, with hypertension as the exposure and intestinal flora as the outcome. It was designed to investigate whether hypertension causes imbalances in the specific intestinal flora. IVs, instrumental variables; LD factor, linkage disequilibrium factor; MR-PRESSO, MR Pleiotropy RESidual Sum and Outlier.

The study used the two-sample bidirectional MR design that assessed the cause-effect interaction between gut flora and hypertension with single nucleotide polymer-phisms (SNPs) as an instrumental variable. Three criteria need to be met for SNPs: associated with exposure, with no confounding of the exposure-outcome association, and affecting outcome only through exposure (27). Figure 1 illustrates the flow chart of the overall process. We used STROBE-MR as the reporting specification for reference (28).

### 2.2. Ethical review

An existing ethical clearance from the respective institutional review board is available for all published genome-wide association studies (GWAS). Only summary-level data were used in this study, where no additional ethical approval was required.

### 2.3. Data sources

All data used in this MR study are publicly available. GWAS data for gut microbes were derived from 16S rRNA gene sequencing profiles and genotyping data curated and analyzed by the MiBioGen International Consortium (29). The study population was mainly adults or adolescents of European origin from 11 countries, with a total sample of 18,340 individuals (24 cohorts). We accessed data on 211 gut microbial taxa at 5 taxonomic levels. Published GWAS of hypertension from the UK Biobank (id: UKB-b-12493) was then used as a data source for outcome events in 463,010 participants of European descent (30). A total of 54,358 patients with hypertension, defined as the presence of essential (primary) hypertension (*International Classification of Diseases 10 diagnosis code I10*) and 408,652 individuals without primary hypertension or any other hypertensive disorder, were included in the study.

### 2.4. Genetic instruments extraction

We chose SNPs up to the genome-wide significance threshold (*P* < 5 × 10^−8^) as instrumental variables (IVs). Because the number of SNPs that met the criteria was too small, we selected IVs with a threshold of *P* < 1 × 10^−5^ to obtain more comprehensive results. To ensure the independence of each SNP, we set the linkage disequilibrium (LD) factor r^2^ to 0.01 and the clumping window width kb = 5,000. Then the information on SNPs related to the above intestinal flora was extracted from the summary GWAS data of hypertension, removing the missing SNPs, and the MAF was put at 0.01. In addition, all SNPs with palindromic structures were excluded to prevent the effect of alleles on the results. F value was used to examine the presence of bias in the causal relationship between intestinal flora and hypertension due to weak IVs. The used SNP is considered a weak IV and deleted when the F-statistic was less than 10.

### 2.5. Statistical analyses

All MR analysis was conducted using R software (version 4.2.1) by the “Two Sample MR” package (https://mrcieu.github.io/TwoSampleMR). *P* < 0.05 was accepted as a statistically significant result for MR analysis. Wald method was used to estimate the causal relationship between exposure and outcome for the bacterial taxa with only one instrumental variable. We utilized MR analysis for gut microbial taxa with multiple IVs using inverse-variance-weighted (IVW), weighted median, and MR-Egger. Fixed or random-effects IVW method was applied as the primary method for MR analysis. Cochrane's Q test was performed to assess the heterogeneity of IVs. If heterogeneity was present (*P* < 0.05), the random-effects IVW test was used to estimate the MR statistic. Since the IVW method requires all SNPs to be valid instrumental variables, its results would be subject to large errors in the presence of invalid IVs. While the significant results of the Weighted median method are still applicable as long as at least 50% of the IVs are valid instrumental variables. Among the three major MR assumptions, MR studies' results are subject to inaccuracy once the assumption is violated that IVs are associated with other confounders in addition to exposure factors. Horizontal pleiotropy was used to weigh the effect. Intercept terms of MR-Egger were one of the most important methods to evaluate the existence of horizontal pleiotropy among IVs. Horizontal polymorphism exists when the intercept is not zero, of which the statistical significance will be determined by the *P*-value of the intercept. Together with MR-Egger, the MR pleiotropy residual sum and outlier method (MR-PRESSO) were also commonly used to detect horizontal pleiotropy. MR-PRESSO consists of the following three main steps, detecting the presence of pleiotropy, calculating the corrected predicted values after removing outliers, and comparing whether the difference between the two predicted values before and after correction is statistically significant. Finally, MR results were evaluated for robustness using a leave-one-out analysis.

As shown by an increased ratio of intestinal Firmicutes/Bacteroidetes in spontaneously hypertensive rats, the dysbiosis can be rebalanced after oral administration of minocycline (31). Indeed, there are changes in the balance of intestinal flora species depending on environmental conditions and individual status. To investigate whether hypertension leads to an imbalance of specific intestinal flora, a bidirectional MR analysis was performed with hypertension as exposure and intestinal flora as the outcome. SNPs with a threshold of *P* < 1 × 10^−5^ were screened as instrumental variables to obtain more comprehensive MR analysis results. Meanwhile, in the bidirectional MR analysis, we gave particular attention to the role of hypertension on the causal effect of the identified statistically significant flora as reverse MR analysis.

## 3. Results

### 3.1. Effect of intestinal flora on hypertension

#### 3.1.1. Selection of IVs

In the genetic analysis of the bacterial taxa on hypertension, a total of 2,427 SNPs were selected as IVs associated with 211 bacterial taxa by removing SNPs with linkage disequilibrium and the presence of palindromic structures with a locus-wide significance level (*P* < 1 × 10^−5^) as the threshold, including 9 phyla (108 SNPs), 16 class (194 SNPs), 20 order (237 SNPs), 35 families (427 SNPs), and 131 genera (1,461 SNPs). The F-statistics of IVs were all greater than 10, suggesting no bias caused by weak instrumental variables (Supplementary Table S1). A series of 32 SNPs were identified as IVs (genome-wide significance, *P* < 5 × 10^−8^), and Supplementary Table S2 specifies the information on these IVs.

#### 3.1.2. Two-Sample MR analysis of intestinal flora taxa to hypertension

**Figure 2:**
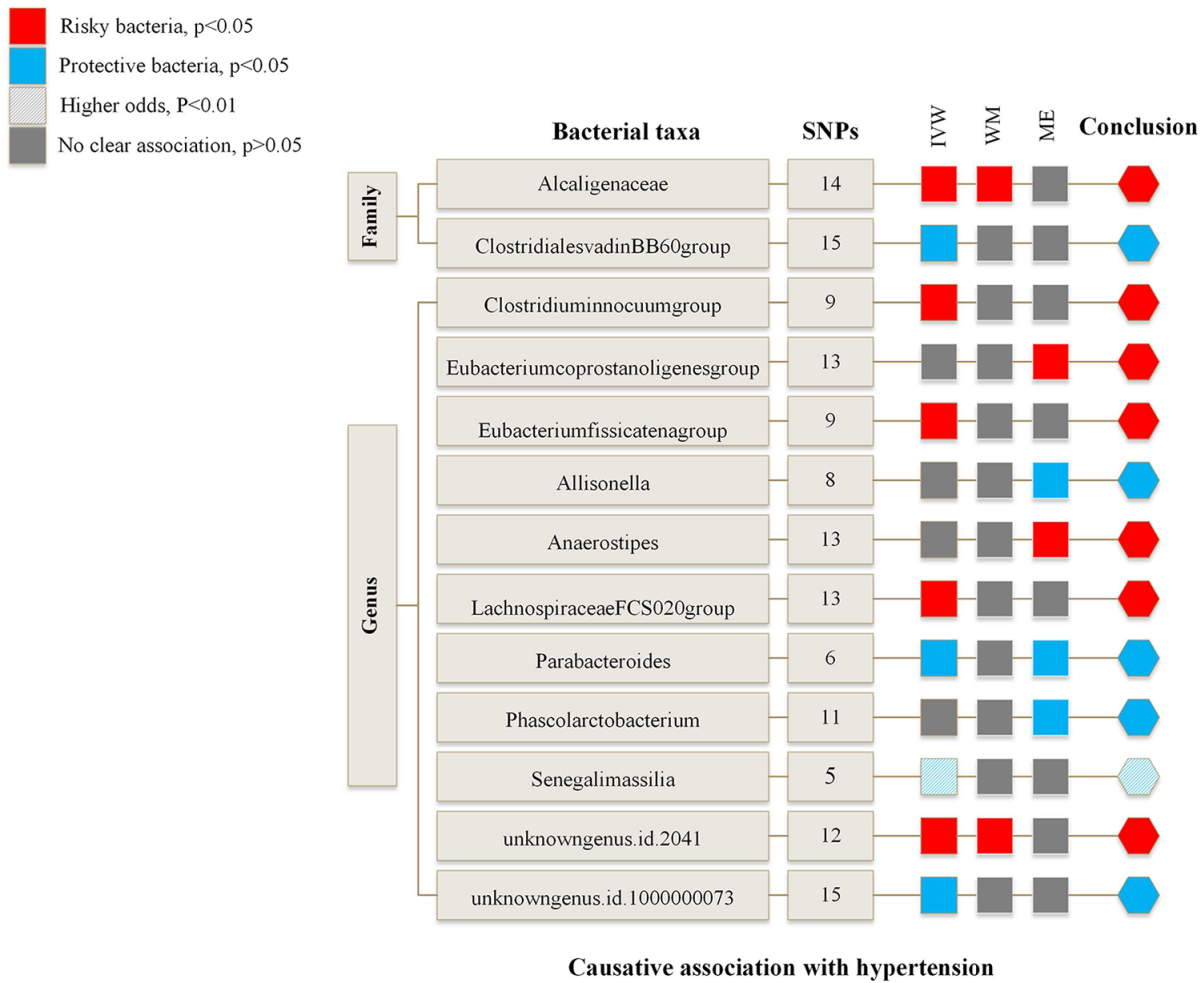
Risk and protective gut microbial genera underlying hypertension—an overview of primary results from the two-sample MR-study. Full data for all results depicted in the figure can be found in the Supplementary. IVW, inverse variance weighted method; WM, weighted median estimator; ME, MR Egger.

Figure 2 is an overview of the primary results from the Two-Sample MR study. It presents that 13 bacterial taxa were statistically significant in MR analysis with hypertension on locus-wide significance level, where 2 were family part and 11 were genera part. Among them, *Alcaligenaceae*, *Clostridiuminnocuum*, *Eubacteriumcoprostanoligenes*, *Eubacteriumfissicatena*, *Anaerostipes*, *LachnospiraceaeFCS020*, *unknowngenus* (id.2041) were risk factors concerning hypertension, *ClostridialesvadinBB60*, *Allisonella*, *Parabacteroides*, *Phascolarctobacterium*, *Senegalimassilia*, and *unknowngenus* (id.1000000073) were protectors for hypertension. In addition, *Senegalimassilia* was more statistically significant in terms of protection as its *P* statistic was less than 0.01.

Supplementary Table S3 and Supplementary Table S4 summarize the results of the MR study and sensitivity analysis for all genera. IVW results indicate that *Alcaligenaceae* [odds ratio (OR) = 1.007; 95% confidence interval (CI), 1.000–1.014], *Clostridiuminnocuum* (OR = 1.005; 95%CI, 1.000–1.009), *Eubacteriumfissicatena* (OR = 1.005; 95%CI, 1.001–1.009), *LachnospiraceaeFCS020* (OR = 1.006; 95%CI, 1.000–1.012), *unknowngenus* (id.2041) (OR = 1.006; 95%CI, 1.001–1.011) were positively correlated with hypertension; *ClostridialesvadinBB60* (OR = 0.994; 95%CI, 0.989–0.999), *Parabacteroides* (OR = 0.987; 95%CI, 0.978–0.997), *Senegalimassilia* (OR = 0.990; 95%CI, 0.983–0.997), *unknowngenus* (id.1000000073) (OR = 0.994; 95%CI, 0.989–0.999) were negatively related to hypertension. The *P* statistics of heterogeneity regarding the above bacterial taxa were all >0.05, and no evidence of horizontal pleiotropy was found by MR Egger. Further assessment of pleiotropy with MRPRESSO found no outliers, indicating the results' reliability with IVW.

MR Egger reveals that there was a relatively greater increase in hypertension among participants with bacterial taxa including *Eubacteriumcoprostanoligenes* (OR = 1.042; 95%CI, 1.007–1.079), *Anaerostipes* (OR = 1.032; 95%CI, 1.008–1.056), while *Allisonella* (OR = 0.952; 95%CI, 0.918–0.987) and *Phascolarctobacterium* (OR = 0.968; 95%CI, 0.941–0.995) were in a negative correlation with hypertension. Besides, MR Egger shows there was horizontal pleiotropy (*P* < 0.05) between the IVs of these four genera and the outcome. We assumed the results of the MR Egger method were more reliable in the above four genera. The pleiotropy of *Eubacteriumcoprostanoligenes* and *Allisonella* was also confirmed by MRPRESSO. However, there was no significant change in results after removing outliers.

**Figure 3:**
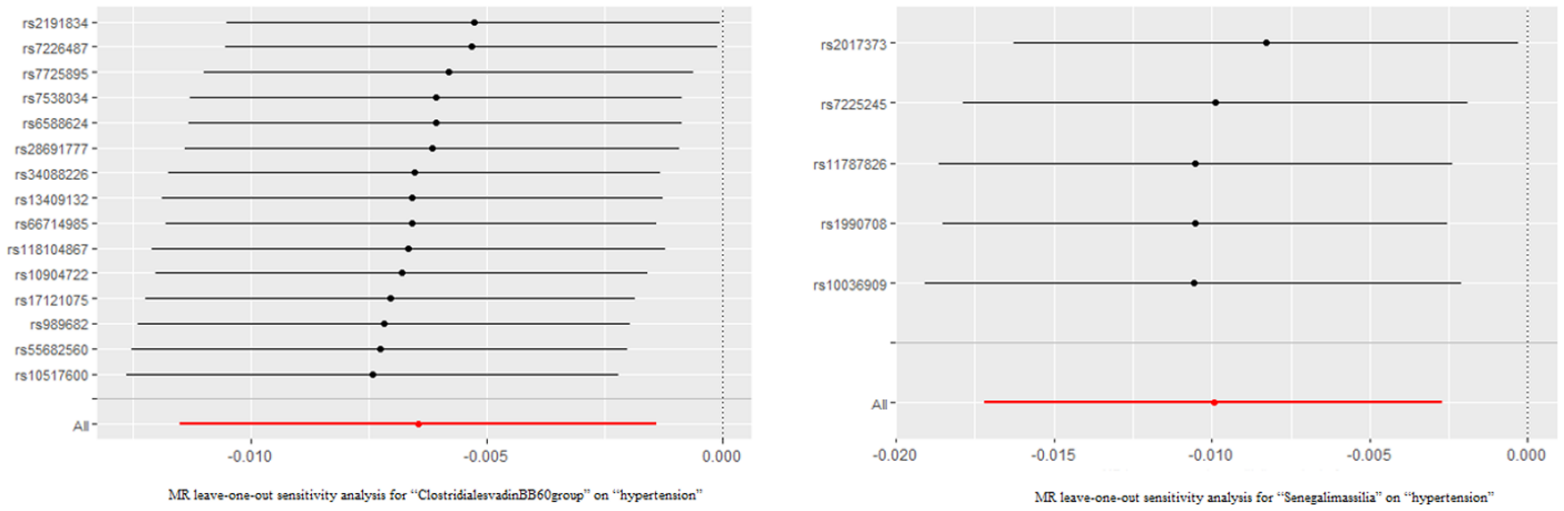
The leave-one-out analysis results of clostridialesvadinBB60 and senegalimassilia. Red lines represented the MR results of IVW analysis, and there was no significant difference in the causal estimation between the two bacteria and hypertension after removing either IV.

The outcomes of the MR study and sensitivity analysis with genome-wide significance threshold are described in Supplementary Table S5 and Supplementary Table S6. Wald ratio indicates that the *Eubacteriumcoprostanoligenes* (OR = 1.026; 95%CI, 1.005–1.048) was a hazard element for hypertension, which is consistent with the previous results of MR analysis at locus-wide significance level. Leave-one-out sensitivity test demonstrated no remarkable difference in the estimation of the causal roles of ClostridialesvadinBB60 and Senegalimassilia on hypertension no matter which IV was excluded, accounting for the robustness of the MR results (Figure 3). The leave-one-out analysis results of the 13 bacterial taxa with statistical significance in MR analysis were displayed in Supplementary Figure S1.

#### 3.1.3. Reverse MR analysis

To prevent reverse causality from interfering with the above results, we performed a reverse MR analysis with significant gut flora in Two-sample MR studies as the outcome and hypertension as the exposure on locus-wide significance level (Supplementary Table S7). Supplementary Figure S2 depicts the reverse MR results for the 13 genera, where elevated BP was negatively correlated with *Phascolarctobacterium* (OR = 0.113, 95%CI, 0.018–0.724). There was no additional evidence for a causal effect of hypertension on the other 12 genera.

### 3.2. Modification of intestinal flora by hypertension

**Figure 4:**
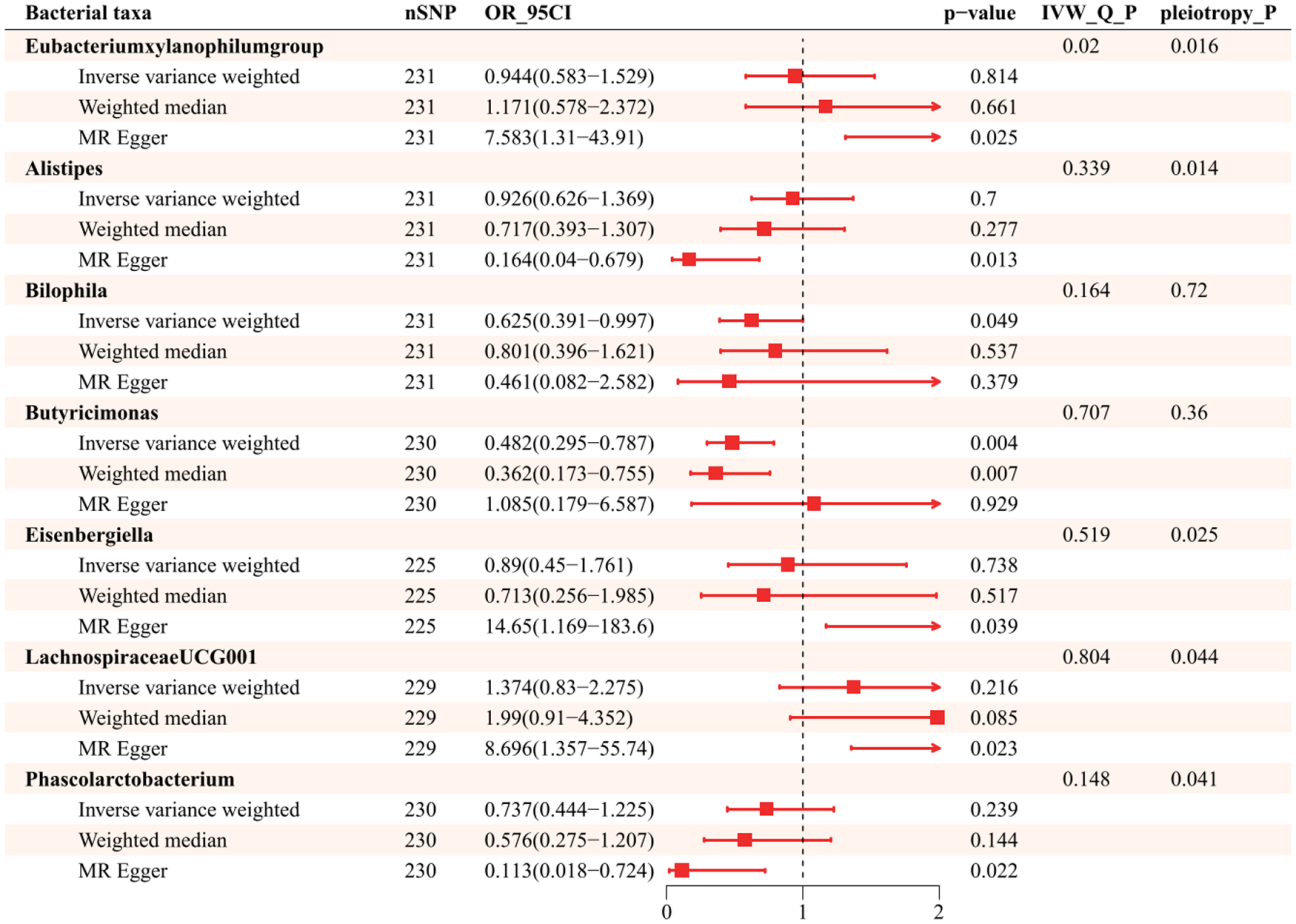
Causality of Hypertension to gut microbiota (*P* < 1.00E-05).

Further exploration of the causal relationship of hypertension on bacterial taxa is summarized in Figure 4. It illustrates all statistically significant results of bidirectional MR analysis for a total of 7 genera causally affected by hypertension. There was horizontal pleiotropy among *Eubacteriumxylanophilum*, *Alistipes*, *Eisenbergiella*, *LachnospiraceaeUCG001,* and *Phascolarctobacterium* (id.2168), which pleiotropy *P* statistics were less than 0.05 (Supplementary Table S8). In this light, we used MR Egger to identify the causal effect of hypertension on these bacterial taxa. MR Egger indicates that those increased attributable to hypertension are *Eubacteriumxylanophilum* (OR = 7.583; 95%CI, 1.310–43.906), *Eisenbergiella* (OR = 14.648; 95%CI, 1.169–183.606), and *LachnospiraceaeUCG001* (OR = 8.696; 95%CI, 1.357–55.736), while the opposite was true for *Alistipes* (OR = 0.164, 95%CI, 0.040–0.679) and *Phascolarctobacterium* (id.2168) (OR = 0.113; 95%CI, 0.018–0.724). IVW suggests that hypertension was negatively associated with *Bilophila* (OR = 0.625, 95%CI, 0.391–0.997), *Butyricimonas* (OR = 0.482; 95%CI, 0.295–0.787) (Supplementary Table S9), and neither heterogeneity nor pleiotropy was significant for these 2 genera (Supplementary Table S10).

## 4. Discussion

Hypertension is a modifiable risk factor for cardiovascular disease development and adverse prognosis (32). Although numerous studies have shown that lifestyle and environmental factors are important drivers for the induction of hypertension, a substantial proportion can nevertheless be explained by genetics (33, 34). Previous large cross-sectional studies have shown significant differences in the overall abundance of gut microbes, and some specific flora in hypertensive patients compared to normotensive individuals (35, 36). From data on hypertension levels using 463,010 UK Biobank participants, we used bidirectional MR analysis and found that 13 gut microbial taxa at 2 classification levels influence hypertension risk, whereas individuals in a hypertensive state could induce alterations in the abundance of 7 intestinal flora. Our study used genetic instrumental variables to demonstrate that onset of hypertension and gut microbiota shifts are mutually causal.

The rapid development of macrogenomics and various sequencing technologies, such as 16S rRNA gene amplicon sequencing and shotgun metagenome sequencing, has allowed researchers to analyze variations in the gut microflora of hypertensive and normotensive populations with great ease (37). We selected the study, which was organized and analyzed by the MiBioGen International Consortium, as the source of data related to the human gut microbiome (29). It provided the largest host genomics and microbiome-based data across multiple races to date and examined the impact of methodological differences on microbiome data. Although the statistical power of microbiome-wide analysis was diminished due to microbiome heterogeneity and inter-individual variability, there still provided a favorable basis for exploring the causal relationship between bacterial taxa and disease traits. In numerous studies, a consistent finding is that lower microbial abundance and diversity or dysbiosis are associated with higher BP (10, 25, 31). An increased *Firmicutes*/*Bacteroidetes* ratio has widely been recognized as an indicator of intestinal ecological dysregulation, and, interestingly, this also applies to hypertension (31, 38). There was a positive association between higher systolic BP and intestinal flora composition with a higher abundance of genera *Klebsiella*, *Anaerotruncus*, *Porphyromonas*, *Eubacteriumsiraeum*, *Actinomyce*, *Prevotellabivia*, *Clostridium cluster IV*, *Streptococcus*, *Eggerthella*, and *Sporobacter*, etc., while bacteria such as *Roseburia*, *Bifidobacterium*, *Coprococcus*, *Synergistetes*, and *Butyrivibrio,* etc. were reduced in hypertensive patients (10, 18, 21, 25, 39, 40). However, the changed genus may not be the candidate intestinal strain affecting hypertension. Currently, relevant studies are yet to be explored in-depth on the exact correlation and specific mechanisms.

Succeeding studies were undertaken in terms of gut flora products to identify the gut flora associated with causing hypertension. Specifically, SCFAs are potentially involved in BP homeostasis (18, 25). Prospective ambulatory BP monitoring found that higher levels of SCFAs such as acetate, butyrate, and propionate in the stool followed higher BP (41). Also, decreased bacterial taxa of butyrate production were observed in rats with obstructive sleep apnea-induced hypertension (42). The major butyrate-producing bacteria in the intestine include *Faecalibacterium*, *Ruminococcaceae*, *Coprococcus*, and *Roseburia* (43, 44). The results of *Clostridiaceae* and *Phascolarctobacterium* as beneficial factors may be relevant to their butyrate secretion (45). The list of bacteria producing acetate, propionate, and hexanoate is longer, which includes *Parabacteroides* and *Allisonella* mentioned in our MR analysis (46–48). It is believed that these higher rates of SCFAs excretion may have reduced circulating SCFAs uptake and that SCFAs-producing microbiota is usually accompanied by lower BP (19). In the case of spontaneously hypertensive rats (SHR), this idea is supported by experiments in which butyrate injections stimulated the center to lower BP (49). To complicate matters, it is possible that SCFAs do not fully exert a hypotensive effect in BP regulation, depending on the role of different SCFA receptors (50, 51). Identifying these receptors may be important to elucidate the pathogenesis of SCFAs-microbiome and hypertension.

On a different note, the differential analysis findings of gut microbiota associated with hypertension were not always consistent. After matching and comparing 67 hypertensive and 62 corresponding normotensive cases by propensity score, Dan et al. found that *Clostridium sensu stricto*, showed a higher abundance in the hypertension group, and the same results were obtained by Huart et al. (23, 41). While the opposite was found by Verhaar et al. (20). *Bacteroides*, *Clostridiales*, *Oscillibacter*, and *Lactobacillaceae* also show varying performances in diverse studies (10, 18, 21, 23, 52). The discrepancy in results may be attributed to several reasons, starting with the fact that the study may not adjust for important confounding factors in the analysis, such as age, ethnic differences in the recruited population, diet, and medications. Alternatively, the taxonomic level at which studies focus on the flora may differ. The reverse causality might be a factor as well. A lack of evidence is available to determine their causative or improvement role in the development of hypertension for the dysregulated intestinal florae. We choose to cite hypertension as the driver and probe whether any gut flora was subsequently altered. Bidirectional MR analysis displayed a significant increase in the abundance of *Eubacteriumxylanophilum*, in agreement with the findings of Kim et al. (21). At the genus level, there was also an increase in *Eisenbergiella* with *Lachnospiraceae* on account of hypertension, and a decrease in *Alistipes*, *Bilophila*, *Butyricimonas,* and *Phascolarctobacterium*. Certain evidence exists that angiotensin I converting enzyme 2 (ACE2) mutant modifies the gut microbial composition (53). G-protein-coupled estrogen receptor (Gper1), a pro-hypertensive agent, has a similar effect that Gper1^−/−^ rats exhibited significantly lower levels of *Clostridiales* beneath the phylum Firmicutes, implying that hypertension susceptibility genes act on gut microbes (54, 55).

A bidirectional MR approach was used for the first time in our study to investigate the causal relationship between primary hypertension and intestinal flora, not only by performing various sensitivity analyses to evaluate the robustness of the analyses but also to exclude the interference of reverse causality. As described in the above results, 6 beneficial and 7 risky microbiota suggested a potentially causal relationship to hypertension, and higher BP also caused disruptions of 7 microbiota abundance. Although the two cohort studies have a fairly large sample size, only recipients of European ancestry were included in the GWAS data source, discounting the extrapolation of the study results. Theoretical causal interventions exist for some bacteria whose specific mechanisms of action are not yet clear. It requires single gut flora transplantation or more animal experiments to confirm the function of microbial metabolites and their contribution to BP modulation. Our team is also conducting related studies in hopes of finding potential strategic targets to control BP.

## 5. Conclusion

In summary, our comprehensive bidirectional MR analysis supports that intestinal flora and hypertension are mutually causal.

## Data Availability

Publicly available datasets were analyzed in this study. This data can be found here: https://mibiogen.gcc.rug.nl/ UK Biobank (id: UKB-b-12493).
