# Supplementary material for: Causality of gut microbiome and hypertension: A bidirectional mendelian randomization study
Source: Front Cardiovasc Med. 2023 May 4;10:1167346. doi: 10.3389/fcvm.2023.1167346 (PMC10192878; doi:10.3389/fcvm.2023.1167346)

***Supplementary Material***

**Causality of Gut Microbiome and Hypertension: A Bidirectional Mendelian Randomization Study**

Yihui Li †, Ru Fu †, Ruixuan Li, Jianwei Zeng, Tao Liu, Xiaogang Li *,

Weihong Jiang *

To whom correspondence should be addressed:

* Xiaogang Li,

Email: hnxylxg@csu.edu.cn

* Weihong Jiang,

Email: jiangweihongdoc@163.com

† These authors contributed equally to this work and share first authorship.

**Supplementary Figure S1.** The Leave-one-out analysis results of the 13 bacteria with statistical significance in MR studies of gut microbiota to hypertension.

| 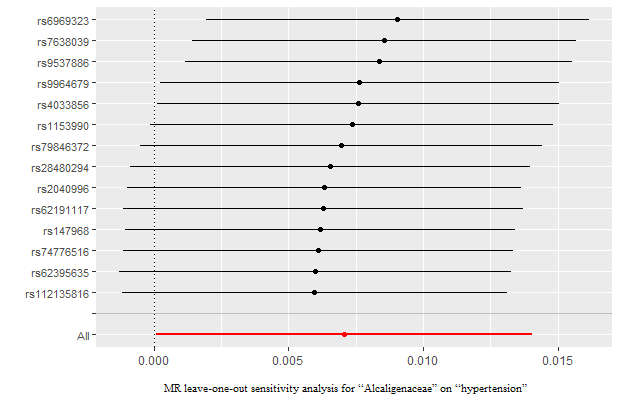 | 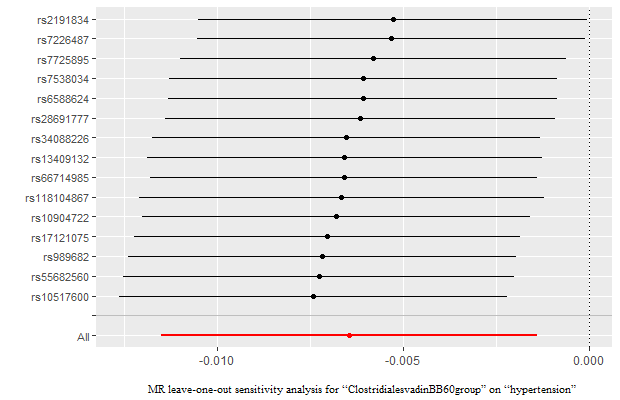 |
| --- | --- |
| 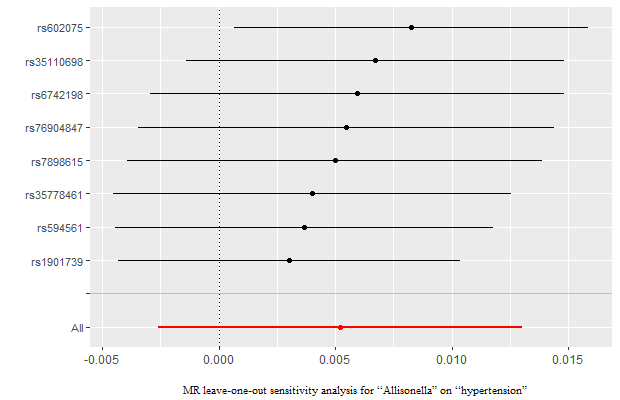 | 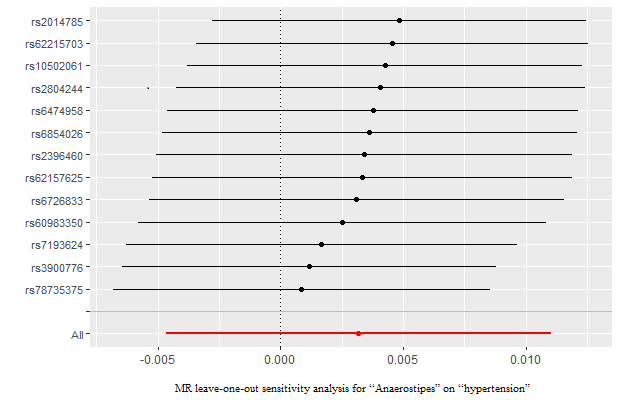 |
| 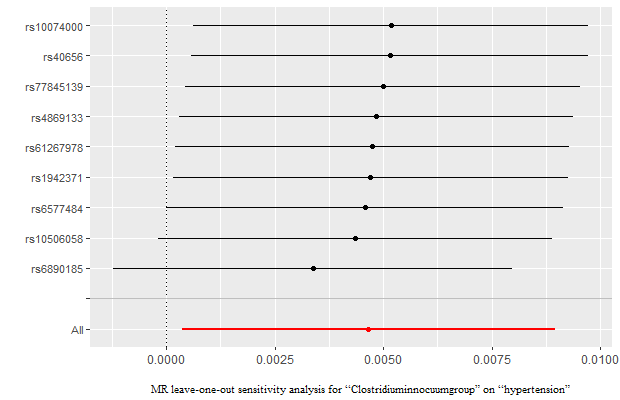 | 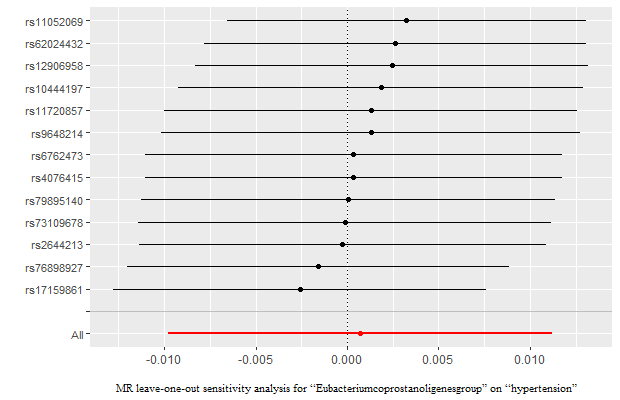 |
| 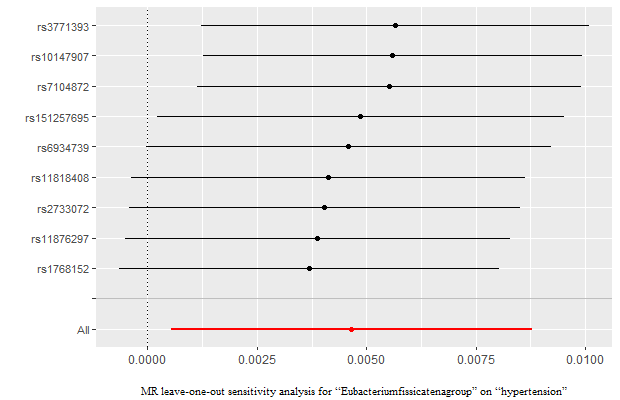 | 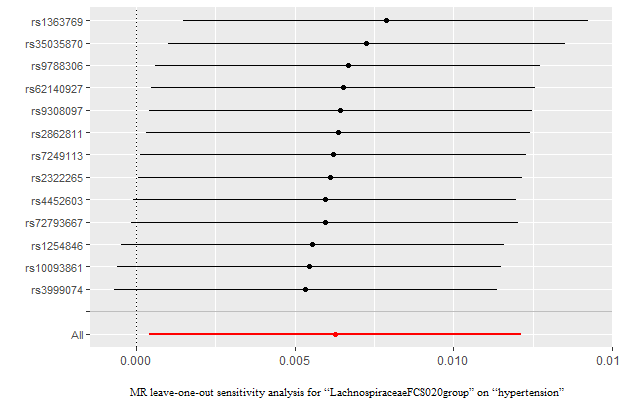 |
| 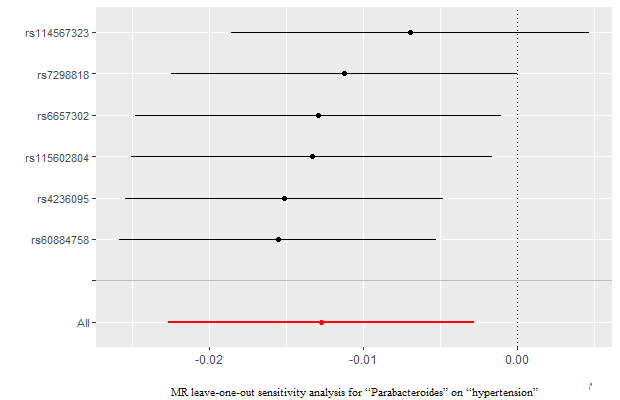 | 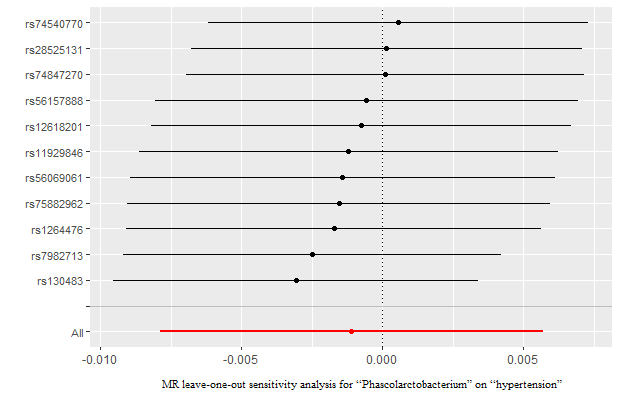 |
| 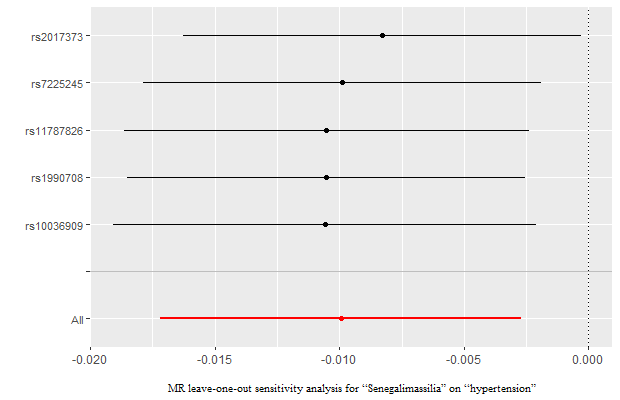 | 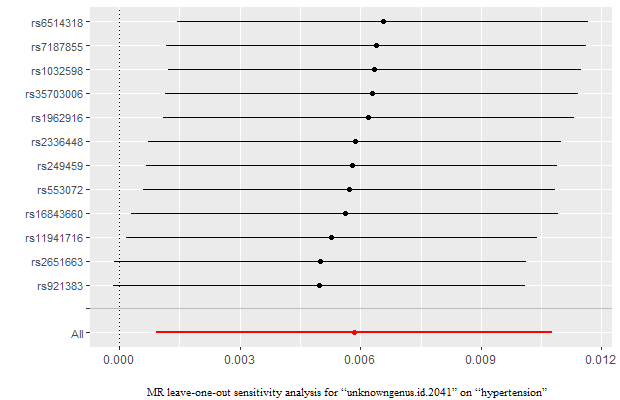 |
| 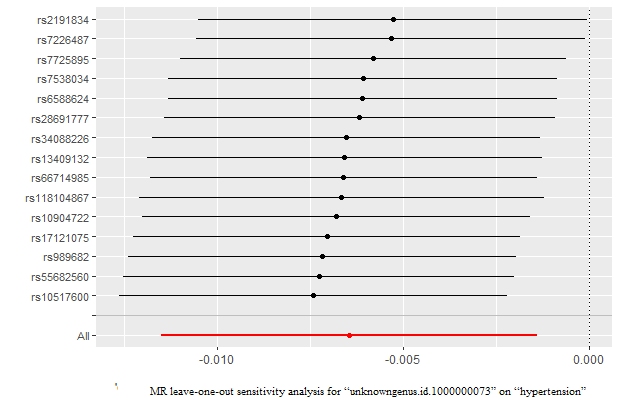 |  |

**Supplementary Figure S2.** Reverse causality inquiry for those florae with statistical significance in MR studies.


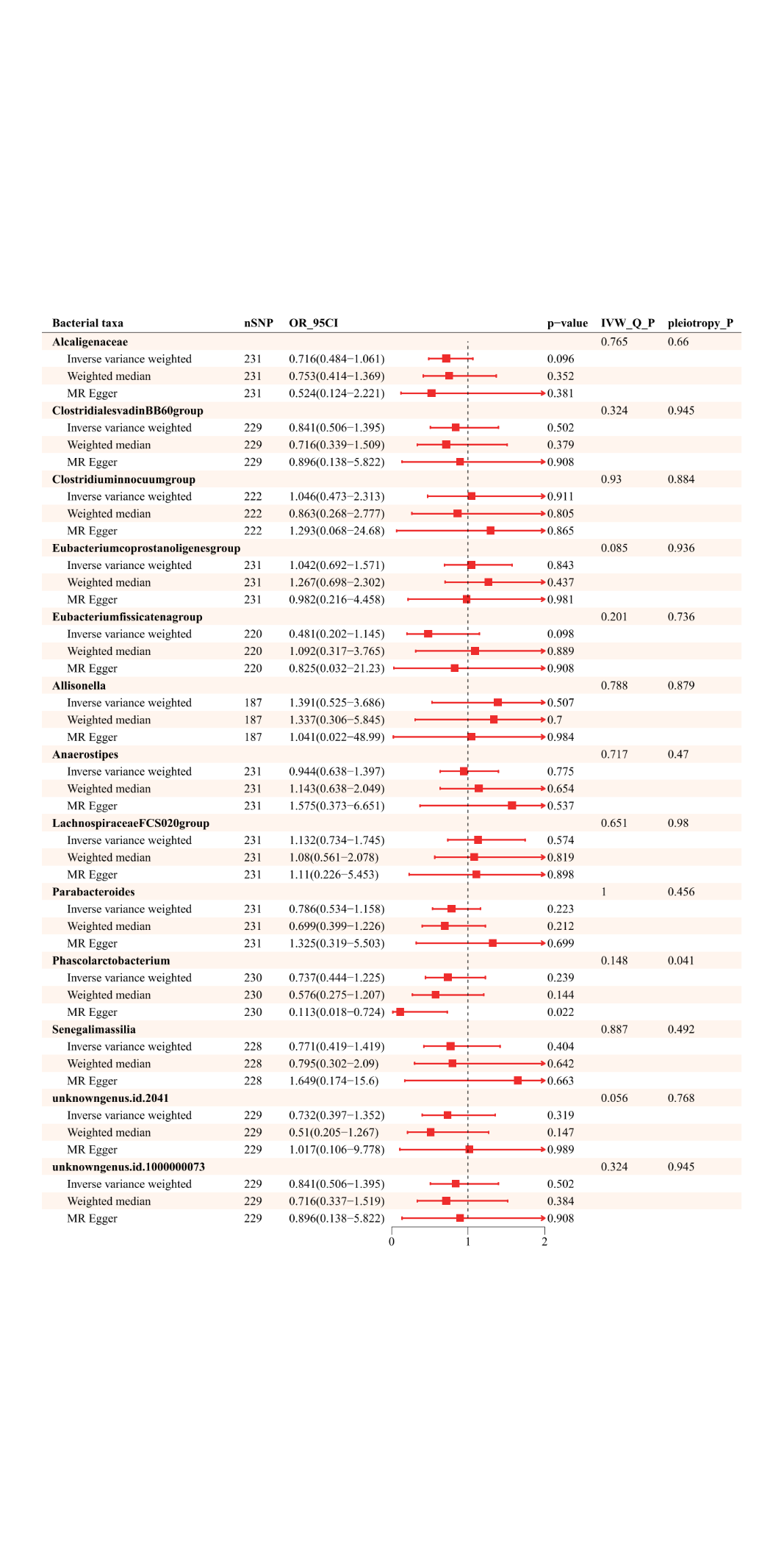

Supplement: Supplementary file 2 [file Datasheet1.docx]
